# Intestinal epithelial replacement by transplantation of cultured murine and human cells into the small intestine

**DOI:** 10.1371/journal.pone.0216326

**Published:** 2019-05-31

**Authors:** Hassan A. Khalil, Sung Noh Hong, Joshua D. Rouch, Andrew Scott, Yonghoon Cho, Jiafang Wang, Michael S. Lewis, Martin G. Martin, James C. Y. Dunn, Matthias G. Stelzner

**Affiliations:** 1 Department of Surgery, UCLA David Geffen School of Medicine, CHS 72–215, Los Angeles, California, United States of America; 2 Division of Gastroenterology and Nutrition, Department of Pediatrics, Mattel Children’s Hospital and the David Geffen School of Medicine at UCLA, University of California, Los Angeles, Los Angeles, California, United States of America; 3 Department of Pathology & Laboratory Medicine, VA Greater Los Angeles Health System, Los Angeles, California, United States of America; 4 Department of Surgery, Stanford University School of Medicine, Stanford, California, United States of America; 5 Department of Surgery, VA Greater Los Angeles Health System, Los Angeles, California, United States of America; University Claude Bernard Lyon 1, FRANCE

## Abstract

Adult intestinal epithelial stem cells are a promising resource for treatment of intestinal epithelial disorders that cause intestinal failure and for intestinal tissue engineering. We developed two different animal models to study the implantation of cultured murine and human intestinal epithelial cells in the less differentiated “spheroid” state and the more differentiated “enteroid” state into the denuded small intestine of mice. Engraftment of donor cells could not be achieved while the recipient intestine remained in continuity. However, we were able to demonstrate successful implantation of murine and human epithelial cells when the graft segment was in a bypassed loop of jejunum. Implantation of donor cells occurred in a random fashion in villus and crypt areas. Engraftment was observed in 75% of recipients for murine and 36% of recipients for human cells. Engrafted spheroid cells differentiated into the full complement of intestinal epithelial cells. These findings demonstrate for the first time successful engraftment into the small bowel which is optimized in a bypassed loop surgical model.

## Introduction

Intestinal failure results from numerous polygenic and monogenic disorders. In its most severe form, it requires life-long parenteral nutrition and/or allogenic intestinal transplantation, both treatments that are associated with several complications [1, 2]. While short-bowel syndrome is the most common cause of intestinal failure, several epithelial disorders result in generalized malabsorption despite a normal small bowel length [1, 3].

Autologous intestinal stem cell (ISC) based therapy may offer an alternative approach [4–6]. Prior work in rats included implantation of fresh organoids consisting of crypts with surrounding mesenchyme into a denuded small intestinal segment [7, 8]. Subsequently, Lahar and coworkers reported that myofibroblasts are essential to enable long-term growth of human small intestinal epithelium in culture [9]. They pre-cultured epithelial cells with added myofibroblasts on biopolymer scaffolds; these seeded scaffolds gave rise to complex mucosal structures when implanted subcutaneously into immunocompromised mice (i.e. as heterotopic engraftments). Orthotopic implantation of colonic epithelial cells in the partially denuded colon has also been described: Recent work focused on engrafting ISC-derived colonoids or enteroids (epithelium without mesenchyme) into a murine model of dextran sulfate sodium colitis [4, 5, 10].

The implanted enteroids constituted a single-layered epithelium containing self-renewing ISCs, progenitor and differentiated lineages. These studies provide preliminary evidence that enteroids could be used as a source for ISC therapy in intestinal disorders. Of note, successful engraftment into the murine small bowel has never been reported. This is primarily due to the technical challenges of denuding and engrafting ISC’s in a small animal model that cannot be fasted for a prolong period of time. In contrast, in the present study, we describe a novel method that leads to successful engraftment into the small intestine of recipient mice of murine and human intestinal mucosal cells that have previously been expanded in *in vitro* cultures.

## Materials and methods

### Animal welfare

All animal studies were approved by the animal research committee at UCLA (ARC #2004–016 and 2012–077). The UCLA animal facility is accredited by the AALAC. This study was carried out in accordance with the recommendations in the Guide for the Care and Use of Laboratory Animals of the National Institutes of Health.

### Procurement of human material

All human tissues used in this study were obtained from de-identified and discarded surgical specimens following clinical surgical pathology evaluation. This was approved by the UCLA Institutional Review Board approved procurement and use of surgical samples, which waived the requirement for informed consent for tissues obtained from the UCLA Translational Pathology Core Laboratory (IRB #11–002504).

### Mouse ISEMF culture

Intestinal subepithelial myofibroblasts (ISEMFs) were isolated from male and female 3–5 week-old C57BL/6 mice and cultured as previously described [11–13]. These were cultured in DMEM with glutaMAX (Invitrogen, Carlsbad, CA), 10% FBS (Invitrogen), and penicillin/streptomycin (Invitrogen) with 20 ng/mL EGF (PeproTech, Rocky Hill, NJ), 0.25 U/mL insulin (Sigma, St. Louis, MO), and 10 mg/mL transferrin (Sigma) in 6-well plates. Once confluent, cells were detached with 0.25% trypsin and 1mM EDTA and serially cultured in T25 or T75 tissue culture flasks. Conditioned medium (CM) was collected from semi-confluent flasks of ISEMFs every 5 days.

### Mouse intestinal crypt culture

Crypts were isolated from 8–12 week-old C57BL/6, C57BL/6-Tg(CAG-EGFP)1Osb/J mice using a previously reported technique [7, 14]. The structures were cultured in a three-dimensional Matrigel matrix in media containing 50 ng/mL EGF, 100 ng/mL noggin, and 500 ng/mL R-spondin-1 alone (“enteroid medium”) or with 1:1 addition of ISEMF-CM and 10 μM Y27632 Rho-kinase inhibitor (“spheroid medium”). Enteroids were mechanically passaged using a 25-gauge needle every 5 days. Spheroids were passaged weekly with enzymatic digestion of the matrix in TrypLE followed by mechanical digestion using a 25-gauge needle.

### Human ISEMF culture

Intestinal subepithelial myofibroblasts (ISEMFs) were isolated human infant small intestine and cultured as previously described [9,13] to obtain conditioned medium. In brief, the ISEMFs were cultured in DMEM with glutaMAX (Invitrogen, Carlsbad, CA), 10% FBS (Invitrogen), and penicillin/streptomycin (Invitrogen) with 20 ng/mL EGF (PeproTech, Rocky Hill, NJ), 0.25 U/mL insulin (Sigma, St. Louis, MO), and 10 μg/mL transferrin (Sigma) in 6-well plates. Once confluent, cells were detached with 0.25% trypsin and 1mM EDTA and serially cultured in T25 or T75 tissue culture flasks. Conditioned medium (CM) was collected from semi-confluent flasks of ISEMFs every 5 days.

### Human intestinal and crypt culture

Crypts were isolated and cultured as previously described in a three-dimensional Matrigel matrix in media containing 50 ng/mL EGF, 100 ng/mL noggin, 1 μg/mL R-spondin-1, and 100 ng/mL FGF10 [14, 15]. To make human enteroid culture medium, 10 mM nicotinamide, 2.5 μM CHIR99021, 10 μM SB202190, and 500 nM LY2157299 TGFβ kinase inhibitor were added and the medium mixed 1:1 with Wnt3a-CM; for human spheroid culture, 10 μM Y27632 Rho-kinase inhibitor was added and the medium mixed 1:1 with human ISEMF-CM. Culture wells were passaged every 5 days. Human intestinal crypts were initially cultured as spheroids, transduced with a GFP expressing lentivirus and frozen at –80°C [14]. Prior to implantation, the spheroids were thawed and cultured in human spheroid culture medium and enteroid culture medium, respectively. Five days later, when these cells had typical spheroid or enteroid phenotypes, they were used for implantation.

### Preparation of 3 D cell cultures for implantation

For all studies, cultured enteroids or spheroids were released from Matrigel through a three-minute incubation with TrypLE at 37°C, mixed with equal volume of 10% FBS and washed twice with HBSS then suspended in a 1:5 dilution of Matrigel (BD Bioscience, San Jose, CA) with HBSS and instilled intraluminally. Donor organoids were used for implantation immediately after harvest and preparation.

### Mouse models and surgical techniques

Two days prior to surgery, 12–20 weeks old male and female C57BL/6J mice or NOD-scid IL2Rgnull (NSG) mice (Jackson Laboratory, Bar Harbor, ME) were placed on a gel diet (ClearH2O, Portland, ME), oral antibiotic solution consisting of trimethoprim-sulfamethoxazole (Hi-Tech Pharmacal Co., Amityville, NY) mixed 1:100 with drinking water, and raised wire floors (Ancare, Bellmore, NY). Mice were weighed, anesthetized with inhaled isoflurane, and given a preoperative subcutaneous injection of ceftriaxone (Hospira, Lake Forest, IL) and buprenorphine SR (Zoopharm, Windsor, CO).

Their ventral abdominal wall was shaved and cleansed with betadine and alcohol swabs. Mice were placed on a warming blanket, then sterilely draped. Through a midline laparotomy and using saline-moistened sterile cotton tipped applicators, a segment of midgut was identified. The cell implantations were performed into jejunal segments that were in continuity or jejunal segments that were bypassed (for details see below). The abdominal wall and skin were then closed in interrupted fashion with 6–0 Prolene and 8–0 Vicryl sutures, respectively. Mice were recovered from anesthesia and kept on oral antibiotic solution for five days. On post-operative day 1, gel diet was restarted. Animals were maintained on raised wire floors throughout their post-operative course. Mice were sacrificed on post-operative day 7 and post-operative day 28, respectively.

#### In-continuity model

**Fig 1:**
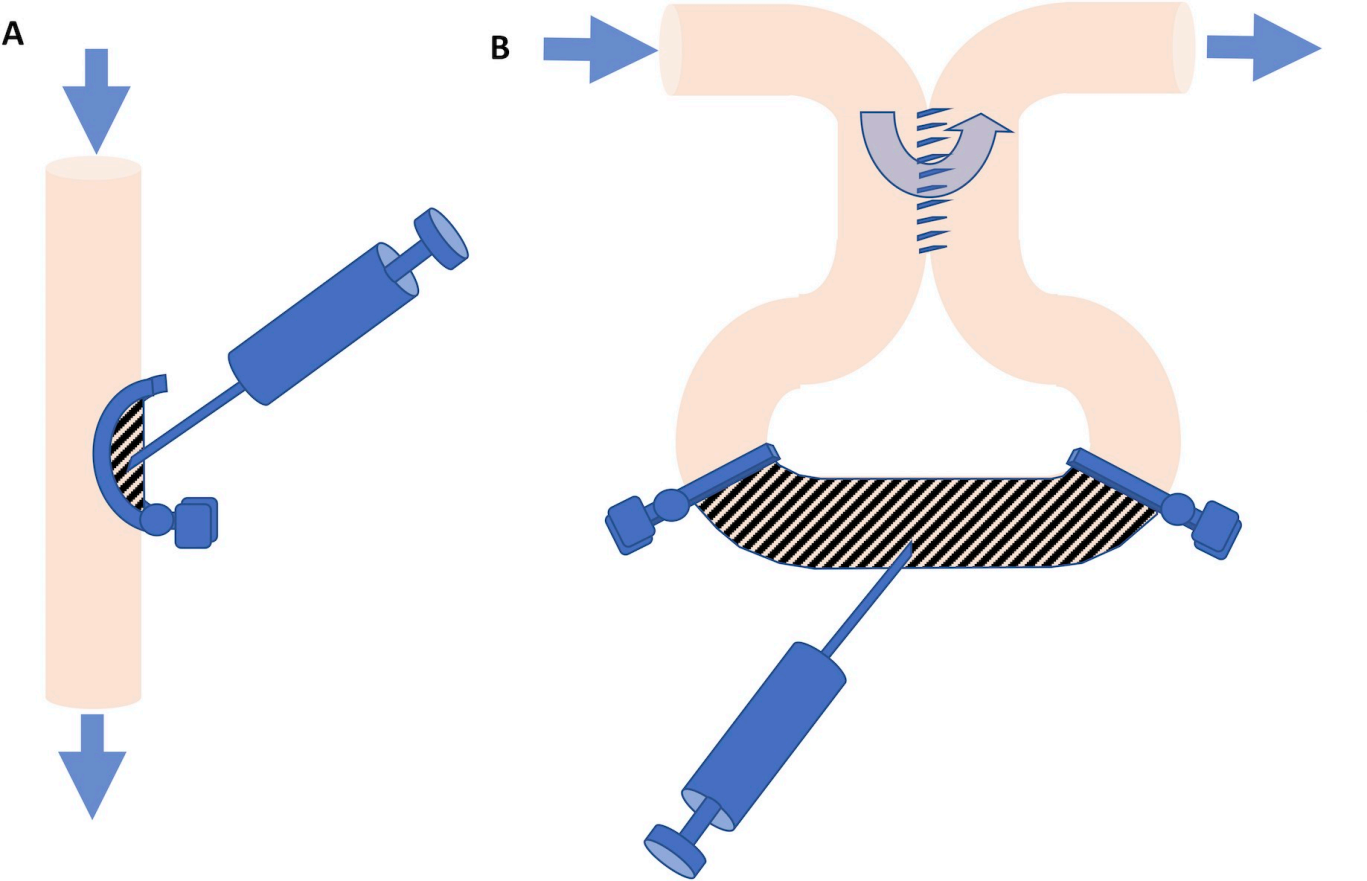
(A) Cells were implanted into a jejunal segment (hatched area) that was in continuity of the small intestine by isolating a mucosal pocket using a semicircular clamp. (B) Alternatively, cells were injected into a bypassed jejunal segment that did not receive the regular stream of bowel content (hatched area). The arrows show direction of the flow of the ingesta.

To denude epithelium from a segment of bowel, a 1 cm segment of midgut was identified and the antimesenteric aspect of the segment clamped with a curved microvascular clamp to create an isolated pocket (Fig 1A). To remove of the resident epithelium, we modified a method we previously described [7]. Into the pocket, 25 μL of a denuding solution consisting of 10 mM ethylenediaminetetraacetic acid (EDTA) and 1 mM dithiothreitol (DTT) in phosphate-buffered saline was injected via a 25-gauge needle. After a 2-minute incubation period, the segment was gently massaged between two moist cotton tipped applicators and the segment was flushed with 5 mL normal saline followed by equal volume of Hanks’ balanced salt solution containing magnesium and calcium (S1A Fig). GFP^+^ mouse organoids, cultured enteroids or cultured spheroids were subsequently implanted using an 18 gauge needle. To retain injected implanted cells temporarily on the luminal side of de-epithelialized segment, TISSEEL fibrin sealant (Baxter, Deerfield, IL) was applied in the lumen of the surrounding intestine. Following implantation, the segments were not flushed. The clamps were removed. The injection site was closed and marked with a stitch of 7–0 Prolene suture.

#### Bypass model

A 5 cm segment of bowel was exteriorized and an 8 mm side-to-side entero-enteric anastomosis was performed in interrupted fashion using 9–0 Nylon sutures creating a 5 cm bypass loop of bowel (Fig 1B). Within this segment, a 2-cm segment was partially denuded of mucosa. To do this, the proximal limb of the bypass loop and the mesenteric vessels supplying the 2 cm segment were successively clamped with atraumatic 10g mini-bulldog clamps or temporarily tied with 7–0 Prolene sutures (S2C Fig). Through a 4 mm distal enterotomy, 200 μL of denuding solution was instilled intraluminally with a mini-bulldog clamp applied proximal to the enterotomy to keep the denuding solution within the segment. After a 2-minute incubation period, the segment was gently massaged between two moist cotton tipped applicators (S2D Fig) and the segment was flushed with 5 mL normal saline followed by equal volume of Hanks’ balanced salt solution containing magnesium and calcium (HBSS; S2E Fig) as in the in-continuity model. Cultured enteroids or cultured spheroids were then injected into the denuded section at a dose of about 2500–4000 cells per recipient using an 18 gauge needle. The mesenteric and proximal clamps were sequentially removed after the cell injection. The ends of the segment were constricted with 8–0 Vicryl sutures to limit inflow of enteric contents but allow passage of mucus out of the segment. The enterotomy was closed in running fashion with 9–0 Nylon suture. The bowel was returned to the peritoneal cavity before the abdomen was closed.

### Histology and immunohistochemistry

Upon retrieval of surgical samples, the lumen of the bypass segment was flushed with cold saline, filled with prewarmed Histogel (American MasterTech, Lodi, CA) and fixed in 10% neutral buffered formalin overnight then stored in 70% ethanol until further histologic processing. Specimens were embedded in paraffin and sectioned at 3 μm thickness. Hematoxylin and eosin (H&E) staining and immunohistochemistry (IHC) were performed per standard protocol [10,12,14]. Staining was done using antibodies against αSMA (Dako, Carpinteria, CA and Abcam, Cambridge, MA), GFP (Aves Labs, Tigard, OR), CDX2 (Dako), E-cadherin (Dako), lysozyme (Abcam), chromogranin A (Immunostar, Hudson, Wisconsin), synaptophysin (Dako), Muc2 (Santa Cruz), and CD10 (Dako). For immunofluorescent stains, appropriate secondary antibodies conjugated to Alexa Fluor 488 or 594 (Life Technologies, Carlsbad, CA) were added at 1:200 dilution and nuclei were counterstained with 4',6-diamidino-2-phenylindole (DAPI, Life Technologies).

## Results

**Fig 2:**
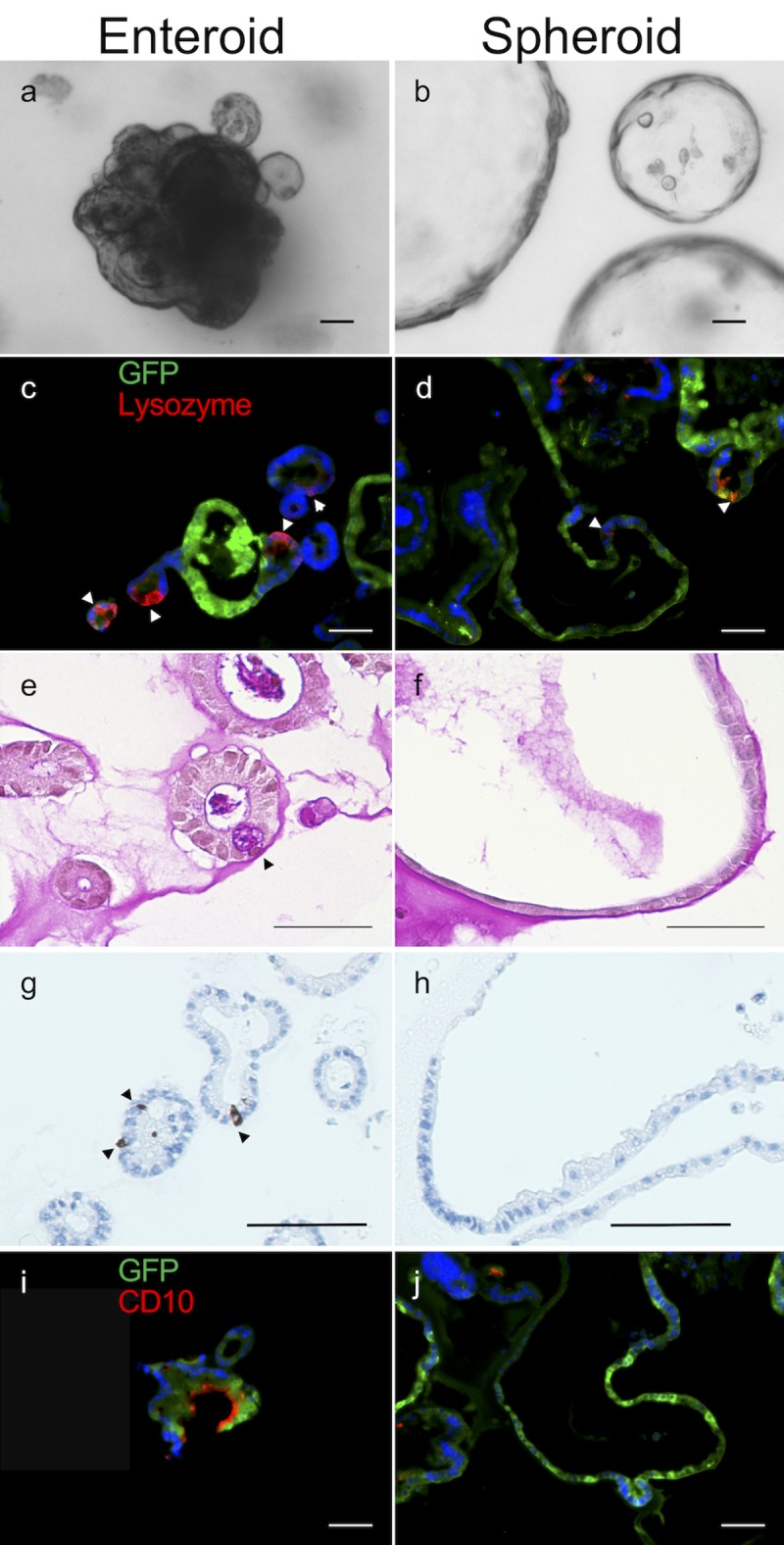
Characterization of murine enteroids and spheroids. (a-b) Brightfield images in culture; (c-j) immunohistochemical analysis, (c-d) lysozyme, (e-f) PAS, (g-h) synaptophysin, (i-j) CD10. The majority of the tested differentiation markers was not visualized in spheroids. Stained structures were obtained from a GFP mouse and showed expected variability in GFP expression. Scale bars, 50 μm.

Using previously reported methods [9, 13, 14], we cultured murine crypts either in traditional culture media to produce “enteroids” [15], or in intestinal subepithelial myofibroblast-conditioned medium (ISEMF-CM) to produce “spheroids” [9]. While epithelium in the enteroids had many features of naturally occurring intestinal crypts, cells forming the spheroidal structures resulting from the latter growth conditions showed minimal evidence of differentiated lineages as assessed by immunohistochemistry (Fig 2).

### Implantation of murine cells

Adult C57BL/6 recipient mice were used as graft recipients in two different experimental models that were described above. In one group, we de-epithelialized a semi-circumferential segment of the intestine wall in the mid-gut region (“in-continuity model,” Fig 1A and S1A Fig). In another group, we performed an entero-enteric bypass in the midgut and de-epithelialized the mucosa in the bypassed segment (“bypass model”, Fig 1B and S2A–S2E Fig). In both models, our de-epithelialization methods produced intestinal luminal areas devoid of mucosal epithelial cells with exposed mucosal mesenchyme while maintaining a relatively intact submucosal architecture (S2F and S2G Fig). Cultured enteroids or spheroids from C57BL/6-GFP mice were implanted onto the de-epithelialized areas in each group of animals, the bowel returned into the abdomen and the laparotomy closed. All animals survived the immediate postoperative period.

Seven-day survival of the recipient mice in the in-continuity model group was 17/20 animals (85%) (Table 1). However, in the “in-continuity” model, we found no engraftment of GFP cells when we assessed the engraftment areas on post-implant day 7 (*n* = 20, including 5 implanted with crypts, 5 with spheroids, and 10 with enteroids). Moreover, while numerous material and various types of implanted cells were used to facilitate engraftment in this model, none led to discernible implantation and growth of GFP^+^ mucosal epithelium (Table 1).

**Table 1:** Synopsis of all implantation experiments. All surviving recipient mice were sacrificed and examined on postoperative day 7 except two mice implanted with spheroids that were sacrificed and examined after 28 days (see asterisks).

Implant Method	Implanted Cells	Number of Mice	Mice Survived	Engraftment Successful
In continuity	Murine crypts	5	4	-
In continuity	Murine spheroids	5	5	-
In continuity	Murine enteroids	10	8	-
Bypass segment	Murine spheroids	7	4*	3**
Bypass segment	Murine enteroids	6	4	3
Bypass segment	Human spheroids	9	5	2
Bypass segment	Human enteroids	9	6	2

*Four mice were alive on postoperative 7; two of these mice were sacrificed that day and one was found to have engrafted cells.

**Two of the four mice alive on postoperative day 7 were sacrificed on postoperative day 28 and both were found to have engrafted cells.

**Fig 3:**
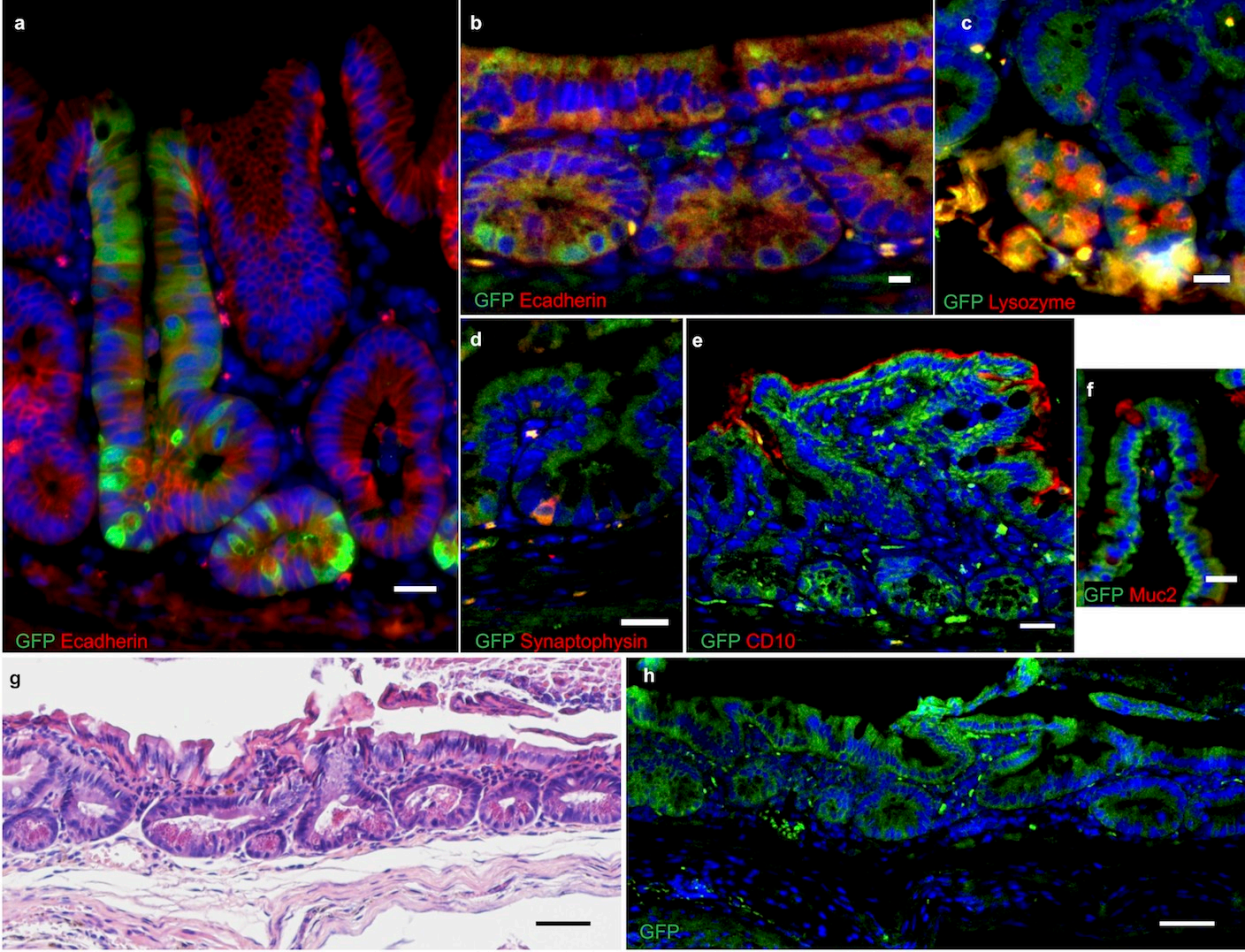
Immunofluorescence stains of engrafted GFP murine spheroids in wild type mouse small intestine. (a) one week after implantation; (b-f) four weeks after implantation: (b) E-cadherin, (c) lysozyme, (d) synaptophysin, (e) CD10, (f) Muc2. (g-h) H&E stain and GFP immunofluorescence of consecutive slides of engrafted mouse intestine four weeks post-implantation. Nuclei are counter-stained with 4',6-diamidino-2-phenylindole (DAPI). Scale bars, 20 μm (a-f) and 50 μm (g-h).

In the bypass model group, six mice received donor enteroids, seven mice spheroids. Three mice died and another two had to be sacrificed; thus 8 of 13 mice were alive on postoperative day 7 (i.e. a survival rate of 62%) (Table 1). GFP-expressing enteroids were found to be successfully engrafted in 3 of 4 recipients (75%) sacrificed on post-operative day 7. Engrafted cells were in juxtaposition to the α-SMA-positive mesenchymal layer. They formed serial invaginations that appeared to represent early crypt-villus architecture. We confirmed the epithelial identity of these cells with E-cadherin staining. Engrafted epithelium showed evidence of differentiation into the full assortment of differentiated lineages characteristic of small intestinal mucosa (Fig 3).

When cells from spheroid cultures were implanted into recipients, we found that they also successfully engrafted in 3 of 4 recipients (75%) sacrificed at one-week post-implantation. Double-immunofluorescence of the transplanted intestinal segment showed cells staining for GFP and E-cadherin (Fig 3A). GFP-expressing cells were observed to differentiate into all intestinal epithelial lineages (Fig 3B–3F). In two animals that had been implanted with spheroids, we assessed the long-term viability of the resulting neo-mucosa and the cellular engraftment persisted for four weeks after implantation (Table 1). Based on our histologic examination, the engrafted cells appeared identical to normal intestinal mucosa (Fig 3G and 3H).

### Implantation of human cells

Cultured human enteroids and spheroids showed the same differentiation patterns that we had observed under identical growth conditions previously [9, 16]. Enteroids contained E-cadherin, Lysozyme, Chromogranin A and Muc2-positive cells; spheroids showed no differentiation markers but were Lgr5-positive. Using the same surgical approach, we implanted human enteroids and spheroids in immune-deficient NSG mice as recipients. Since the “in-continuity” model have not led successful engraftment using murine cells, we employed only the entero-enteric bypass model in the human cell engraftment studies. In order to establish the feasibility and outcome of the procedure, an entero-enteric bypass was performed in 12 mice, of which 6 (50%) survived to post-implantation day 7. The other six either were found in poor health and sacrificed per experimental protocol (n = 4) or were found dead (n = 2).

**Fig 4:**
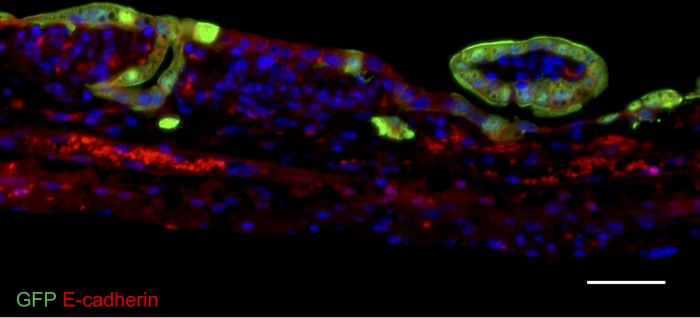
Immunofluorescence stain of engrafted GFP human enteroids in NSG mouse small intestine one week after implantation with anti-E-cadherin antibodies. Nuclei are counter-stained with DAPI. Scale bar, 50 μm.

**Fig 5:**
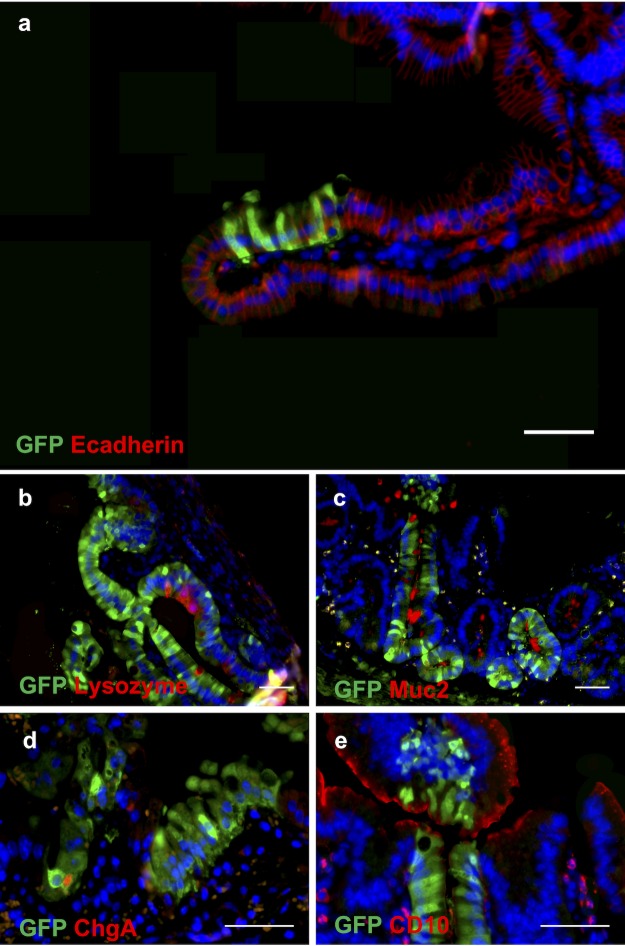
**Immunofluorescence stains of engrafted human GFP spheroids in the small intestine on post-implantation week 1**. **(a)** E-cadherin, **(b)** lysozyme, **(c)** Muc2, **(d)** chromogranin A and **(e)** CD10. Nuclei are counterstained blue with DAPI. Scale bars, 50 μm.

We then engrafted a consecutive series of 18 mice and ultimately had total of 11 mice that had survived to postoperative day 7. We found the positive engraftment of cultured human cells was detected in 4 of these 11 mice. (Spheroids, n = 2; enteroids, n = 2; total engraftment rate = 36%; Table 1; Figs 4 and 5).

We examined those animals that did not survive the first postoperative week. However, it was not possible to determine with certainty before postoperative day 7 if an actual implantation of GFP cells had occurred due to the significant histologic alteration of the mucosa that resulted from the de-epithelialization procedure, the subsequent healing process and the scattered GFP signals emitted by non-engrafted cell fragments.

In both murine and human cell implantations, the engraftment of individual cells appeared to be a random geographic event. At seven days post-transplantation, the engrafted cells repopulated the surface of the intestine anywhere between the crypt base and the villus tip (see Figs 3 and 5).

## Discussion

With recent advances in ISC expansion and CRISPR/Cas9 editing [15–18] ISC-based therapy for epithelial disorders using ablation and engraftment techniques such as those presented here may become possible. Investigators have grown small intestinal and colonic mucosal cells successfully on biodegradable scaffolds in an attempt to generate a substitute mucosa in *in vivo* engraftment models [19]. In contrast, implantation of intestinal epithelial cells onto a native graft bed in the intestine has rarely been described. Only in the colon, the proof-of-concept has been demonstrated by transplanting cultured murine colonic epithelium into mice with DSS-induced colitis with restoration of the colonic epithelial barrier function and reversal of weight loss [5]. A more recent study described successful engraftment of human colonic organoids into recipient mice after careful chemical and mechanical debridement of the murine colon wall [10]. In contrast, there have been no studies demonstrating implantation of intestinal mucosal cells into the native small intestine, or engraftment of intestinal mucosal epithelium that is derived from humans into any area of the intestine.

We have previously shown the formation of a neo-epithelium upon the heterotopic implantation of biopolymer scaffolds that were seeded with both epithelial and mesenchymal cells and then pre-cultured before implantation resulted formation of a neo-epithelium [9]. However, in the present study, we hypothesized that mesenchyme-free cultured murine and human small intestinal mucosal epithelium can engraft in a small intestinal section whose native epithelium has been removed while leaving behind some native mesenchyme cells below the basement membrane. Our group had previously reported similar studies in which we removed the resident mucosa in a segment of jejunum and seeded the underlying tissue with intestinal organoids that had been prepared fresh from donor mucosa [8, 20]. However, that approach did not involve any *in vitro* expansion of the pool of donor cells and was therefore of limited therapeutic value. Here, we set out to propagate small intestinal mucosal cells *in vitro* first and tested if these cultured cells can be engrafted. We experimented with two phenotypes of epithelial cultures: spheroids–thin-walled, balloon-like structures with lower expression of epithelial differentiation markers, or enteroids containing ISCs at their crypt-like budding extensions and differentiated cells at their villus-like domains [14–16, 21]. We demonstrate that murine enteroids and spheroids are both capable of reconstituting the mucosa in denuded small intestine. The implanted spheroids, despite their more primitive state in culture, reconstituted the small intestinal mucosa and differentiated into all the appropriate cell lineages *in vivo*. The morphologic features resembled observations we make in our previous heterotopic implantation model implanting seeded scaffolds under skin of immunocompromised mice [9].

At one-week post-transplantation, the engrafted cells repopulated the surface of the intestine anywhere between the crypt base and the villus tip. This seems to suggest that engraftment was a random process with implanted cells attaching to the exposed mesenchyme at the first point of contact. While we harvested organoids, spheroids and enteroids carefully from groups of intact multi-cell conglomerates, the preparation and implantation procedure invariably leads to some fragmentation of these delicate structures. This may explain why we observe implantation of cells even half way up the villus in some cases (e.g. Fig 5A).

This study provides a first proof-of-concept that a partially ablated small intestinal epithelium can be successfully repopulated with mesenchyme-free cultured epithelial cells. This methodology could for example be used to convert a jejunal segment that is not capable of active bile acid or vitamin B12 absorption into a substitute ileum by engrafting it with ileal cells [7]. Further additional work is needed to develop a clinically applicable method to denude native small intestinal mucosa.

Our cell transplantation technique led to successful implantation in an entero-enteric bypass model. The simpler in-continuity model proved ineffective. This may be due to the higher shear forces that may act on the freshly engrafted cells while the flow of ingesta is still passing by the transplantation site in the latter model. We attempted to limit this effect by depositing fibrin as a temporary filler material around the engraftment site. However, this proved insufficient to ensure engraftment of donor cells. To advance this field, development of simpler and more effective alternative techniques of transplantation will be required. We previously used biodegradable scaffolds successfully as a graft bed [9]. However, we believe future focus ought to be directed at graft bed optimization in the native intestinal tissues without the use of biopolymers. Yet so far, little is known about the requirements for an optimal graft bed for intestinal mucosal cells in the small intestine. The previously described methods of chemical and mechanical deepithelialization are rather crude and carry a certain mortality risk [10]. Furthermore, in the present study, the NSG mice did not tolerate the bypass operation well and the procedure showed a significant mortality. NSG mice are also not an ideal animal model for surgical engraftment efforts since they are more likely to succumb to infectious complications of intestinal surgery due to their immune deficiency. In addition, current culture techniques require supplementation of recombinant growth factors and any clinical application of this transplantation technique in human patients will depend on development of an allogen-free culture system. Finally, the highly variable effects of the denudation and of the engraftment in different areas were a shortcoming of this study. We estimate that the engraftment efficiencies for human enteroids and spheroids were under 5% of the denuded mucosal surface area, respectively. However, it would be desirable to quantify the efficiency of the engraftment rates for enteroids and spheroids more precisely. This would require extensive morphometric studies which should be added to future investigations.

## Conclusion

This study provides a first proof-of-concept that a partially ablated small intestinal epithelium can be successfully repopulated with mesenchyme-free cultured murine and human mucosal epithelial cells. Further additional work is needed to develop a clinically applicable method to denude native small intestinal mucosa.

## Supporting information

S1 Fig In-continuity model. (a) Denuding of a hemi-circumferential segment of small bowel; (b) H&E stain of denuded small bowel one week later. The denuded site had been marked with a large suture (arrow) during the denudement a week prior. The entire epithelium is here in a state of regeneration. (c-d) absence of positive staining for GFP protein at the engraftment site, thus no evidence for engraftment of GFP enteroids one week after transplantation.
(TIFF)

S2 Fig Entero-enteric bypass model. (a) Loop of ileum exteriorized via midline laparotomy, (b) entero-enteric anastomosis, (c) temporary ligation of mesenteric vessels and proximal bowel, and clamping of distal bowel to create a 2 cm isolated segment for denuding, (d) mechanical denuding using cotton tip applicators after infusion of denuding solution, (e) flushing denuded segment. (f) H&E section of partially denuded ileum; scale bar, 500 μm, (g) magnified view of boxed region in (f) demonstrating denuded epithelium.
(TIFF)

## Abbreviations

ISC: Intestinal stem cell
ISEMF: Intestinal subepithelial myofibroblast
ISEMF-CM: Intestinal subepithelial myofibroblast-conditioned medium
