# Supplementary figures and images for: Intestinal epithelial replacement by transplantation of cultured murine and human cells into the small intestine
Source: PLoS One. 2019 May 31;14(5):e0216326. doi: 10.1371/journal.pone.0216326 (PMC6544206; doi:10.1371/journal.pone.0216326)

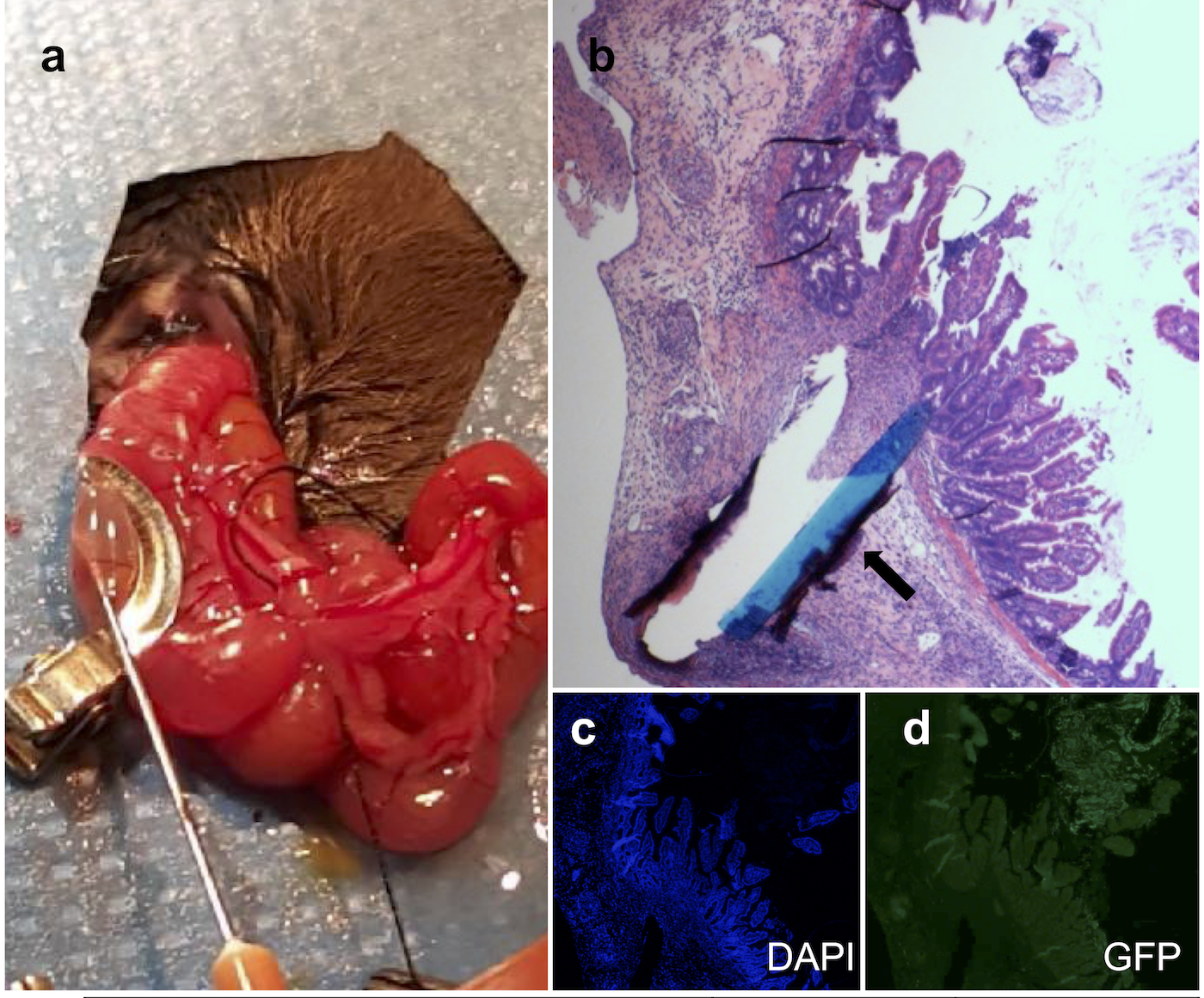

Supplement: S1 Fig — (a) Denuding of a hemi-circumferential segment of small bowel; (b) H&E stain of denuded small bowel one week later. The denuded site had been marked with a large suture (arrow) during the denudement a week prior. The entire epithelium is here in a state of regeneration. (c-d) absence of positive staining for GFP protein at the engraftment site, thus no evidence for engraftment of GFP enteroids one week after transplantation. (TIFF) [file pone.0216326.s001.tiff]

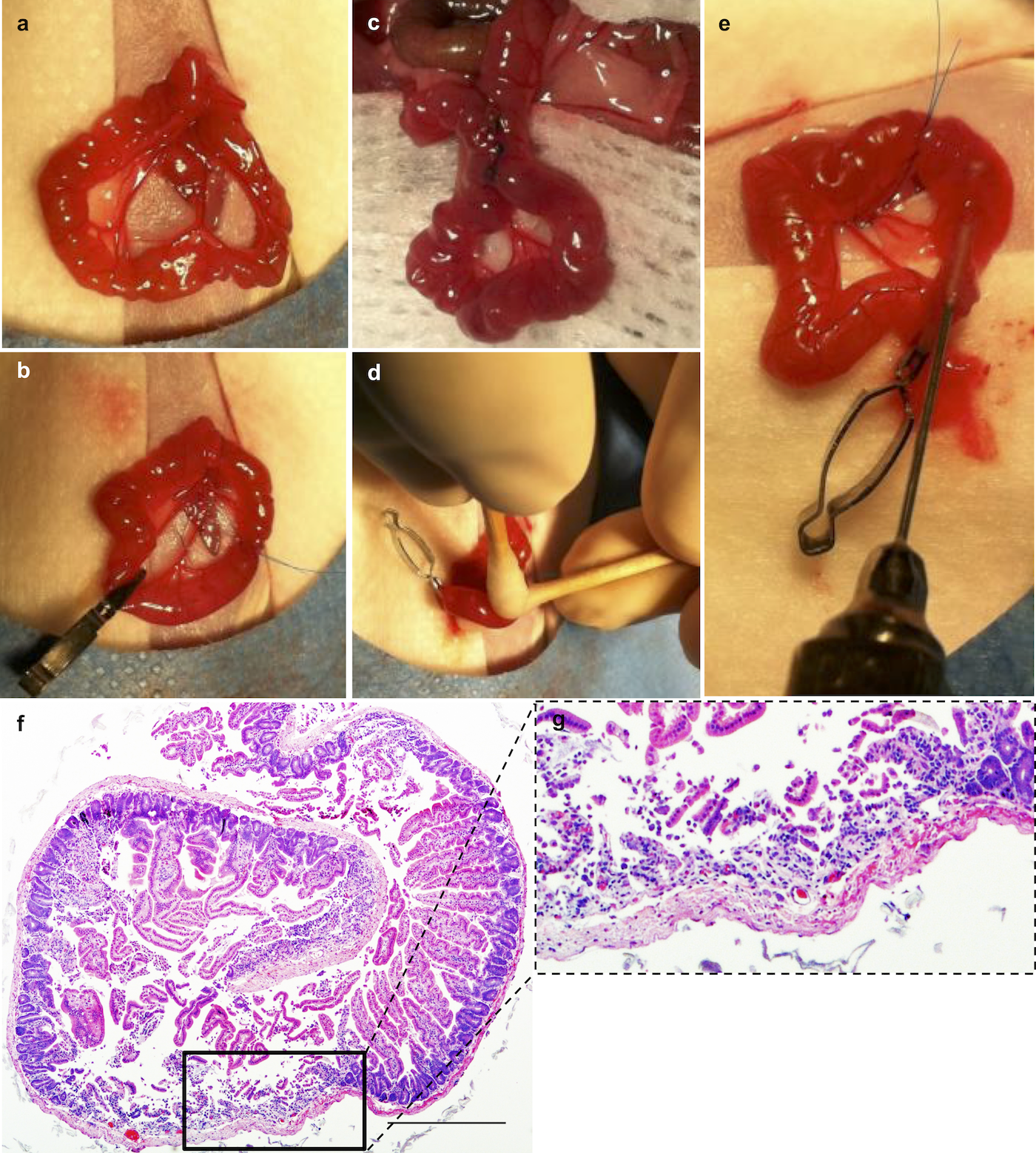

Supplement: S2 Fig — (a) Loop of ileum exteriorized via midline laparotomy, (b) entero-enteric anastomosis, (c) temporary ligation of mesenteric vessels and proximal bowel, and clamping of distal bowel to create a 2 cm isolated segment for denuding, (d) mechanical denuding using cotton tip applicators after infusion of denuding solution, (e) flushing denuded segment. (f) H&E section of partially denuded ileum; scale bar, 500 μm, (g) magnified view of boxed region in (f) demonstrating denuded epithelium. (TIFF) [file pone.0216326.s002.tiff]
